# Effects of Salt Tolerance Training on Multidimensional Root Distribution and Root-Shoot Characteristics of Summer Maize under Brackish Water Irrigation

**DOI:** 10.3390/plants12183329

**Published:** 2023-09-20

**Authors:** Suhan Peng, Tao Ma, Teng Ma, Kaiwen Chen, Yan Dai, Jihui Ding, Pingru He, Shuang’en Yu

**Affiliations:** 1 College of Agricultural Science and Engineering, Hohai University, Nanjing 211100, China; hhupengsuhan@hhu.edu.cn (S.P.); daiyan@hhu.edu.cn (Y.D.); dingjihui@hhu.edu.cn (J.D.); seyu@hhu.edu.cn (S.Y.); 2 Jiangsu Province Engineering Research Center for Agricultural Soil-Water Efficient Utilization, Carbon Sequestration and Emission Reduction, Nanjing 211100, China

**Keywords:** summer maize, brackish water irrigation, root distribution, root-shoot characteristics, salt tolerance

## Abstract

To investigate the impact of brackish water irrigation on the multidimensional root distribution and root-shoot characteristics of summer maize under different salt-tolerance-training modes, a micro-plot experiment was conducted from June to October in 2022 at the experimental station in Hohai University, China. Freshwater irrigation was used as the control (CK), and different concentrations of brackish water (S0: 0.08 g·L^−1^, S1: 2.0 g·L^−1^, S2: 4.0 g·L^−1^, S3: 6.0 g·L^−1^) were irrigated at six-leaf stage, ten-leaf stage, and tasseling stage, constituting different salt tolerance training modes, referred to as S_0-2-3_, S_0-3-3_, S_1-2-3_, S_1-3-3_, S_2-2-3_, and S_2-3-3_. The results showed that although their fine root length density (FRLD) increased, the S_0-2-3_ and S_0-3-3_ treatments reduced the limit of root extension in the horizontal direction, causing the roots to be mainly distributed near the plants. This resulted in decreased leaf area and biomass accumulation, ultimately leading to significant yield reduction. Additionally, the S_2-2-3_ and S_2-3-3_ treatments stimulated the adaptive mechanism of maize roots, resulting in boosted fine root growth to increase the FRLD and develop into deeper soil layers. However, due to the prolonged exposure to a high level of salinity, their roots below 30 cm depth senesced prematurely, leading to an inhibition in shoot growth and also resulting in yield reduction of 10.99% and 11.75%, compared to CK, respectively. Furthermore, the S_1-2-3_ and S_1-3-3_ treatments produced more reasonable distributions of FRLD, which did not boost fine root growth but established fewer weak areas (FLRD < 0.66 cm^−3^) in their root systems. Moreover, the S_1-2-3_ treatment contributed to increasing leaf development and biomass accumulation, compared to CK, whereas it allowed for minimizing yield reduction. Therefore, our study proposed the S_1-2-3_ treatment as the recommended training mode for summer maize while utilizing brackish water resources.

## 1. Introduction

Brackish water irrigation, as an important unconventional water resource utilization method, plays a crucial role in alleviating the contradiction between water supply and demand, which contributes to ensuring food production security [1,2]. In China, the natural replenishment of brackish water reaches 24.6 billion cubic meters, mainly distributed in salt-affected areas with a total area of over 36.67 million ha, serving as crucial backup irrigation water sources for such areas [3,4]. However, the utilization of brackish water for irrigation inevitably increases the soil salt content, which may lead to secondary salinization issues [5]. Therefore, the core issue of using brackish water for agricultural irrigation lies in scientifically exploring the reasonable, safe, and efficient utilization methods of brackish water. 

The alternated or mixed irrigation of brackish and freshwater methods can control the accumulation of salt in the root-zone and alleviate the inhibitory effect on crop growth [6,7,8]. However, in current studies, the applications of such methods were still concentrated in the middle and later stages of the crop cycle [9,10,11], with the irrigation volume of brackish water being less than half of the irrigation quota. This was because current studies generally believed that most crops were more sensitive to salt stress in the early growth stages, and their tolerance gradually increased as the growth stage progresses [12], which was very similar to crops’ response to drought stress [13]. In contrast, numerous studies have demonstrated that subjecting crops to moderate drought stress during certain periods of vegetative growth, under drought stress conditions, could train their stress tolerance and ultimately promote yield formation [14,15,16]. Therefore, in the practicing of brackish water irrigation, further research is needed to see whether it is possible to proactively irrigate the appropriate concentration of brackish water during the salt-sensitive stage, to stimulate rapid improvement in crop salt tolerance and benefit their later growth stages, which is similar to the training effect of deficit irrigation.

The root system is the first organ to come into contact with and be affected by the toxic effects of excessive salt ions in the soil [17]. The length, volume, total surface area, root vitality, and root turnover of the root system all affect crop water uptake and the accumulation of related nutrients, thereby directly influencing crop growth and development [18,19,20]. Current research on crops such as cotton [21], wheat [22], cowpea [23], and sunflower [24] have shown that when crops were subjected to salt stress during the early growth stages, they preferentially allocated limited dry matter to the root system to ensure water and nutrient uptake. Therefore, a rational brackish water irrigation approach should be able to regulate crop root system growth, especially the growth of active fine roots [25], which can alter the crop’s osmotic regulation ability [26] and dry matter allocation strategy, ultimately enhancing the crop’s salt tolerance and alleviating the inhibitory effects. However, the current focus of most research still lay in the impact of brackish water irrigation on above-ground growth indicators, and insufficient research was found on the fundamental factor of the root system, which could directly alter the crop’s salt tolerance capability.

Therefore, this study selected summer maize, a major food crop, as the research object, and employed different concentrations of brackish water irrigation to create various salt tolerance training modes. The study focuses on the dynamic growth distribution of the multidimensional root system and the root and shoot growth regulation strategies of maize, aiming to clarify the basis of crop salt tolerance variation based on root spatial distribution, and quantifies the enhancement effects of salt tolerance under different training modes. This study will contribute to developing more efficient methods of utilizing brackish water resources and further exploring the water-saving potential in salt-affected areas.

## 2. Results

### 2.1. Changes in Leaf Area Index of Summer Maize at Different Growth Stages

**Figure 1:**
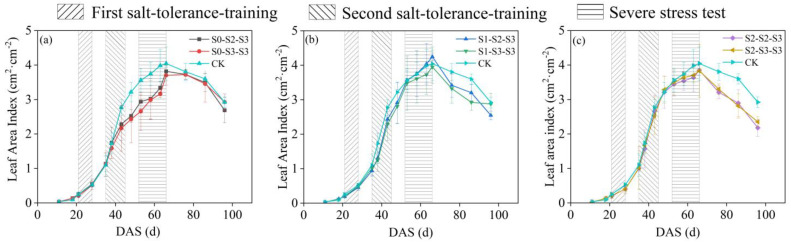
Dynamic changes in leaf area index (LAI) of summer maize under brackish water irrigation during the entire growth period. The data are averaged measurements (n = 4), and vertical bars indicate standard deviation. The S0, S1, S2, and S3 represent different salt concentration levels of irrigation water, corresponding to none (0.08 g·L^−1^), mild (2.0 g·L^−1^), moderate (4.0 g·L^−1^), and severe (6.0 g·L^−1^) stress. DAS is the abbreviation for days after sowing.

Leaf area index (LAI) is an important indicator in field experiments that reflects the growth status of crops. In this study, during the first salt-tolerance-training (FSTT) stage (DAS = 21~28), mild salt stress (S1) had no significant impact on the leaf development of summer maize; however, moderate salt stress (S2) exhibited a certain inhibitory effect on the leaf development of summer maize, with reductions of 23.30% and 23.56% in LAI for the S_2-2-3_ and S_2-3-3_ treatments, compared to the CK treatment (Figure 1b,c). During the second salt-tolerance-training (SSTT) stage (DAS = 35~45), the situation changed. S_0-2-3_, S_0-3-3_, S_1-2-3_, S_1-3-3_, S_2-2-3_, and S_2-3-3_ exhibited reductions of 17.64%, 21.76%, 12.17%, 17.36%, 4.09%, and 8.67%, respectively, compared to CK. During the severe stress test (SST) stage (DAS = 52~66), the LAI of summer maize in all treatments continued to rise. At silking (DAS = 66), except for S_0-3-3_, the LAI reached their maxima for the other treatments, ranking from highest to lowest as S_1-2-3_, CK, S_1-3-3_, S_2-2-3_, S_2-3-3_, and S_0-2-3_. After the silking stage, the leaves of summer maize in all treatments began to gradually senesce, resulting in decreases in LAI. Among them, the rates of decrease in LAI for S_0-2-3_ and S_0-3-3_ were lower than that for the other treatments. Additionally, the time for LAI to reach its maximum was later for S_0-3-3_, compared to the other treatments (Figure 1a), but this peak value was still lower than that of the other treatments.

### 2.2. Biomass Accumulation in Summer Maize at Different Growth Stages

#### 2.2.1. Shoot and Root Biomass 

**Figure 2:**
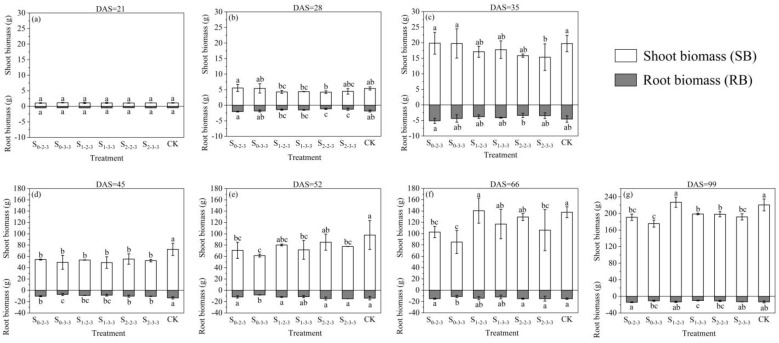
Dynamic changes in shoot biomass (SB) and root biomass (RB) of summer maize under brackish water irrigation during the entire growth period. The data are averaged measurements (n = 4), and vertical bars indicate standard deviation. Different lowercase letters represent significant differences in SB and RB. DAS is the abbreviation for days after sowing. S_0-2-3_, S_0-3-3_, S_1-2-3_, S_1-3-3_, S_2-2-3_, S_2-3-3_ are abbreviations for S0-S2-S3, S0-S3-S3, S1-S2-S3, S1-S3-S3, S2-S2-S3, S2-S3-S3, respectively. The S0, S1, S2, and S3 represent different salt concentration levels of irrigation water, corresponding to none (0.08 g·L^−1^), mild (2.0 g·L^−1^), moderate (4.0 g·L^−1^), and severe (6.0 g·L^−1^) stress.

Biomass is an important indicator for measuring the accumulation of organic matter and nutrient content in plants. As shown in Figure 2, salt stress generally had a significant impact on SB and RB throughout the growth period. For SB, after the FSTT stage (DAS = 21~28), the SB of S_0-2-3_, S_0-3-3_, and CK treatments were obviously higher than the other treatments. After a recovery stage (DAS = 29~34), compared to CK, S_1-2-3_, S_1-3-3_, S_2-2-3_, and S_2-3-3_ resulted in a reduction in SB by 13.38%, 9.99%, 19.69%, and 22.18%, respectively. After the SSTT stage (DAS = 35~45), the SB of all treatments irrigated with brackish water significantly decreased compared to CK (*p* < 0.05) (Figure 2d). Among them, the SB of S_0-2-3_ increased by 9.76% compared to S_0-3-3_, the SB of S_1-2-3_ increased by 9.17% compared to S_1-3-3_, and the SB of S_2-2-3_ increased by 5.34% compared to S_2-3-3_. At the beginning of the SST stage (DAS = 52), compared to those at the start of the SSTT stage (DAS = 35), S_2-2-3_ and S_2-3-3_ exhibited greater magnitude of increase, while S_0-2-3_ and S_0-3-3_ showed smaller magnitude of increase than the other treatments (Figure 2c,e). At the ending of the SST stage (DAS = 66), S_1-2-3_ displayed the highest SB, and compared to those at the beginning of the SST stage, both S_1-2-3_ and S_1-3-3_ had greater magnitude of increase than the other treatments (Figure 2e,f). At harvest (DAS = 99), the SB for S_1-2-3_ and CK were higher than those of the other treatments, with all differences reaching a significant level (*p* < 0.05). S_0-2-3_ and S_0-3-3_ exhibited greater magnitude of increase after the SST stage, but their SB were lower than those of the other treatments at harvest (Figure 2f,g).

For RB, all treatments exhibited a trend of initial increase followed by decrease. Except for S_2-3-3_, which started to decrease before the ending of the SST stage, the RB values for all treatments increased with the number of days after sowing until DAS = 66, but with different magnitudes of increase at different stages. During the FSTT stage, S_0-2-3_, S_0-3-3_, and CK showed much higher increases in RB compared to the other treatments (Figure 2a,b). At the ending of this stage, their RB values significantly differed from S_2-2-3_ and S_2-3-3_ (*p* < 0.05). However, this pattern changed during the SSTT stage, while the S_2-2-3_, S_2-3-3_, and CK treatments exhibited increases of over 190%, greatly higher than the other treatments. S_0-3-3_ only showed an increase of 67.72%, and its RB was the smallest at DAS = 45. During the SST stage, S_0-3-3_ exhibited a higher magnitude of increase compared to the other treatments. However, at the end of this stage, its RB value remained the smallest (Figure 2f). At harvest, all treatments exhibited a decrease in RB to varying degrees, with the RB values in the following order from highest to lowest: S_0-2-3_, CK, S_1-2-3_, S_2-3-3_, S_2-2-3_, S_0-3-3_, S_1-3-3_. This differed obviously from the pattern observed for SB.

#### 2.2.2. Grain Yield

The grain yield of summer maize is a primary indicator for assessing its response to salt stress. As shown in Table 1, different treatments generally exhibited an inverse relationship between grain number per ear and hundred-grain weight. In terms of yield formation, all treatments irrigated with brackish water showed lower grain yields than the CK treatment. Compared to CK, S_0-2-3_ and S_0-3-3_ decreased by 18.74% and 20.17%, respectively; S_1-2-3_ and S_1-3-3_ decreased by 5.98% and 8.61%, respectively; S_2-2-3_ and S_2-3-3_ decreased by 10.99% and 11.75%, respectively. This indicated that for similar irrigation modes, the higher the mineralization degree of the irrigated brackish water, the lower the grain yield. Additionally, S_0-3-3_ and S_0-2-3_ showed the largest decrease, which also had significant differences from the CK treatment (*p* < 0.05).

### 2.3. Dynamic Changes in Multidimensional Root Distribution of Summer Maize

#### 2.3.1. Vertical Distribution of Fine Roots throughout the Entire Growth Period

**Figure 3:**
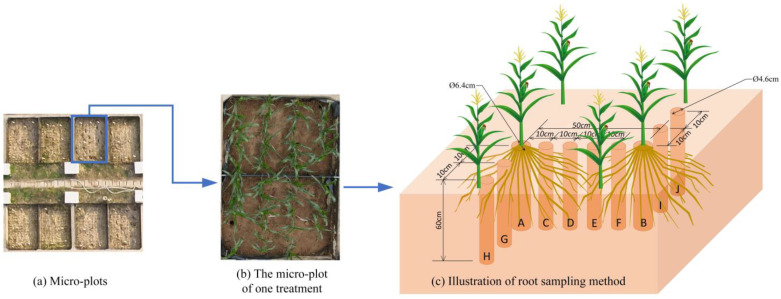
Micro-plots plan and illustration of root sampling method. Positions A and B were designated as sampling locations for the crown, primary, and seminal roots, while positions C–J were designated as sampling locations for the lateral roots. Root sampling was conducted at positions C and G on 21, 28, 35, 45 and 99 days after sowing. On 52 days after sowing, root sampling was conducted at positions A, C, G, and H. Then, 66 days after sowing, root sampling was conducted at all designated positions.

**Figure 4:**
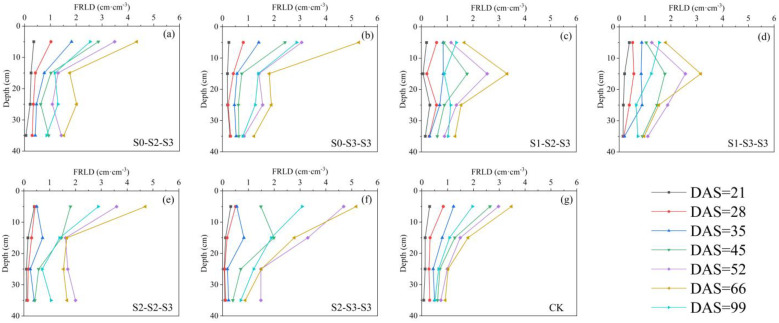
Changes in FRLD at different soil depths during the entire growth period of summer maize under different treatment. The data are averaged measurements at positions C and G in Figure 3. The S0, S1, S2, and S3 represent different salt concentration levels of irrigation water, corresponding to none (0.08 g·L^−1^), mild (2.0 g·L^−1^), moderate (4.0 g·L^−1^), and severe (6.0 g·L^−1^) stress. DAS is the abbreviation for days after sowing. FRLD is the abbreviation for fine root length density.

The root length density is an important metric in root studies. In this study, the roots within 2 mm diameter were counted and then converted into the indicator of fine root length density (FRLD). Micro-plots plan and illustration of root sampling method are shown in Figure 3. A total of seven sampling events were conducted, covering the entire growth period of summer maize. The distributions of FRLD obtained from these seven sampling events are shown in Figure 4. It could be seen from the figure that throughout the entire growth period, the changes in FRLD of S_1-2-3_ and S_1-3-3_ treatments were relatively similar. Their fine roots grew rapidly in the 10~20 cm soil layer and maintained dense distributions in this region since the ending of the SSTT stage (DAS = 45). The other treatments, however, exhibited a different pattern, with their FRLD being highest in the 0–10 cm soil layer. Among them, S_0-3-3_, S_2-2-3_, and S_2-3-3_ treatments even showed boosting trends in their fine root growth. 

After the FSTT stage (DAS = 21~28), the FRLD values in 0~40 cm depth for S_1-2-3_, S_1-3-3_, S_2-2-3_, and S_2-3-3_ decreased by 2.38%, 4.45%, 39.54%, and 49.70%, compared to the CK treatment (Figure 4c–g). This situation underwent a shift during the recovery stage (DAS = 29~34). At DAS = 35, the FRLD in 0~40 cm depth for S_2-2-3_ and S_2-3-3_ were only 1.88 cm^−3^ and 1.80 cm^−3^, respectively, which were much lower than the other treatments. However, compared to those at DAS = 28, their growth rates reached 78.43% and 105.64%, respectively, which were much higher than the other treatments. During the SSTT stage (DAS = 35~45), the CK treatment showed an increase of 76.74% in FRLD in 0~40 cm depth, while the average increase for S_2-2-3_ and S_2-3-3_ was 140.59%, and for S_1-2-3_ and S_1-3-3_ was 67.88%. The average increase for S_0-2-3_ and S_0-3-3_ was only 52.90%. During the SST stage (DAS = 52~66), maize plants transitioned from vegetative growth to reproductive growth. The FRLD of S_2-3-3_ decreased by 5.74% in 0~40 cm depth (Figure 4f), while the FRLD of the other treatments further increased. Among them, the increase of FRLD in 0~40 cm depth for S_0-2-3_ and S_0-3-3_ reached 32.16% and 48.37%, respectively, which were higher than the other treatments. At harvest (DAS = 99), all treatments experienced varying degrees of decline in FRLD. Among them, S_0-2-3_ and S_0-3-3_ showed a decline of 39.10% and 38.06%, respectively, compared to those at DAS = 66, which were lower than the other treatments.

#### 2.3.2. Two-Dimensional Distribution of Fine Roots during the Nutritional Growth Stage 

**Figure 5:**
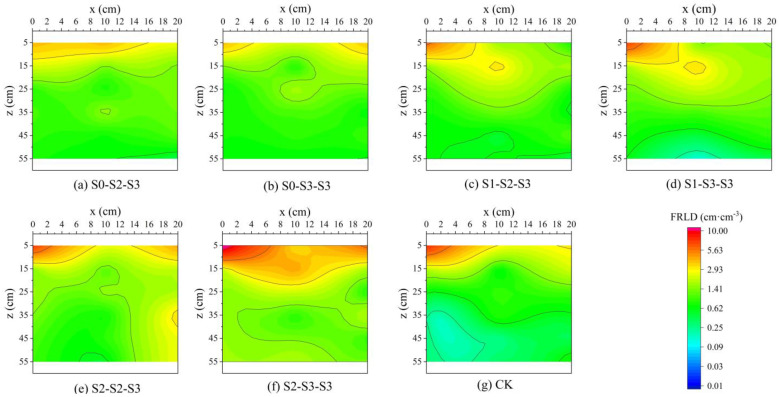
2D distribution of FRLD of summer maize in each treatment on 52 days after sowing. The data are derived from measurements at positions A, G, and H in Figure 3. The S0, S1, S2, and S3 represent different salt concentration levels of irrigation water, corresponding to none (0.08 g·L^−1^), mild (2.0 g·L^−1^), moderate (4.0 g·L^−1^), and severe (6.0 g·L^−1^) stress. FRLD is the abbreviation for fine root length density.

The distribution of FRLD in maize at the tasseling stage, sampled at DAS = 52, are shown in Figure 5. It can be seen from the figure that the FRLD of all treatments were concentrated in 0~20 cm depth. In the horizontal direction, except for the S_2-3-3_ treatment, the FRLD of other treatments were concentrated within a radius of about 10 cm from the plant. Vertically, the FRLD values gradually decreased with increasing depth. Among them, the root extension range of S_2-2-3_ and S_2-3-3_ exceeded that of the other treatments, and their average FRLD in the soil profile increased by 45.40% and 75.48%, compared to the CK treatment, respectively. However, for the FRLD in the 0~20 cm soil layer directly below the plants (x = 0 cm), S_2-2-3_ showed an increase of 34.42% compared to CK, while S_2-3-3_ showed a decrease of 11.52% compared to CK. S_1-2-3_ and S_1-3-3_ exhibited reductions of 26.97% and 10.40%, respectively, in the FRLD within the 0~20 cm soil layer at x = 0 cm, compared to CK. However, both treatments showed relatively dense distributions of FRLD in the 10~20 cm soil layer at a distance of 10 cm from the plant (Figure 5c,d), which were consistent with the vertical distribution in Figure 4c,d. Moreover, compared to the CK treatment, the average FRLD in the soil profile decreased by 5.90% in S_1-2-3_, while it increased by 7.18% in S_1-3-3_. The S_0-2-3_ and S_0-3-3_ treatments exhibited reductions of 36.58% and 47.34%, compared to CK, respectively, in the FRLD within the 0~20 cm soil layer at x = 0 cm, which were much lower than the other treatments. However, both treatments showed elongated root systems, with S_0-2-3_ showing a relatively dense distribution of FRLD in the 30~40 cm soil layer at a distance of 10 cm from the plant (Figure 5a), and S_0-3-3_ showing a relatively dense distribution of FRLD in the 20~30 cm soil layer at a distance of 10 cm from the plant (Figure 5b). Furthermore, compared to the CK treatment, the average FRLD in the soil profile increased by 2.23% in S_0-2-3_, while it decreased by 5.64% in S_0-3-3_.

#### 2.3.3. Three-Dimensional Distribution of Fine Roots during the Reproductive Growth Stage 

**Figure 6:**
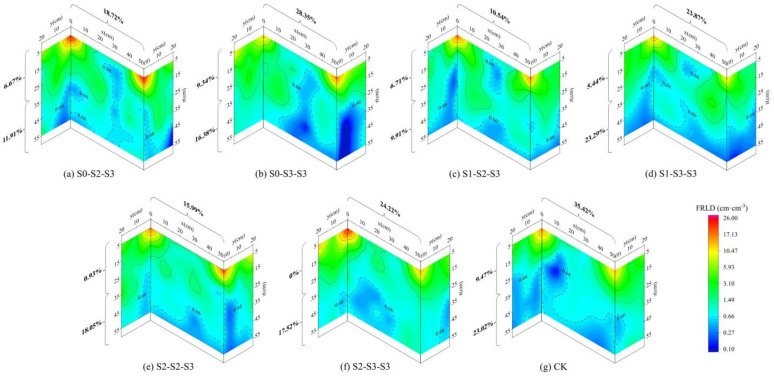
3D distribution of FRLD of summer maize in each treatment on 66 days after sowing. The data are derived from measurements at all designated positions in Figure 3. The S0, S1, S2, and S3 represent different salt concentration levels of irrigation water, corresponding to none (0.08 g·L^−1^), mild (2.0 g·L^−1^), moderate (4.0 g·L^−1^), and severe (6.0 g·L^−1^) stress. FRLD is the abbreviation for fine root length density. The values within the curly brackets on the upper half of the Z-axis indicate the proportion of weak FRLD areas (FRLD < 0.66 cm^−3^) in the 0~30 cm soil layer in the three-dimensional graph. The values within the curly brackets on the lower half of the Z-axis indicate the proportion of weak FRLD areas in the 30~60 cm soil layer in the three-dimensional graph. The values within the curly brackets above the X-axis represent the proportion of weak FRLD areas in the lateral view of the three-dimensional graph.

The distribution of FRLD in maize at the silking stage, sampled at DAS = 66, are shown in Figure 6. In general, over 65% of fine roots was distributed in 0~20 cm depth, and over 87% was distributed in 0~40 cm depth at this stage. Compared to the CK treatment, the average FRLD of 0~60 cm depth increased by 32.26% in S_0-2-3_, 13.89% in S_0-3-3_, 18.54% in S_2-2-3_, and 31.06% in S_2-3-3_, while S_1-2-3_ only increased by 2.35% and S_1-3-3_ even decreased by 9.68%. In addition, when analyzing based on the criterion of FRLD < 0.66 cm^−3^ to identify weak FRLD areas, it was found that the proportions of the weak areas in CK, S_1-2-3_, and S_1-3-3_ were 32.49%, 16.62%, and 28.73%, respectively, indicating more reasonable distributions of FRLD in S_1-2-3_ and S_1-3-3_, compared to CK (Figure 6c,d,g). 

Horizontally, in 0~10 cm depth, all treatments exhibited the highest FRLD directly below the plants (x = 0 cm), and FRLD decreased with increasing distance from the plants. Below 10 cm depth, FRLD might increase and then decrease with increasing distance from the plants, exhibiting dense distribution at certain positions. This trend was more pronounced in the treatments irrigated with brackish water, compared to CK, indicating that irrigation with brackish water promoted horizontal root extension in the soil below 10 cm depth. Vertically, as the soil depth increased, the FRLD of each treatment showed a decreasing trend. Except for a few positions, this top-down decreasing trend was more pronounced in 0~40 cm depth, but there might be a phenomenon of larger FRLD below 40 cm depth. The majority of weak areas in all treatments were located in the 30~60 cm soil layer, particularly in S_2-2-3_ and S_2-3-3_, where the weak areas in the 0~30 cm soil layer were less than 1% (Figure 6e,f).

Moreover, there were great differences in the FRLD distribution between treatments in the inter-row soil. The percentage of weak areas in the inter-row soil of each treatment, from largest to smallest, was CK, S_0-3-3_, S_2-3-3_, S_1-3-3_, S_0-2-3_, S_2-2-3_, S_1-2-3_, with respective values of 35.42%, 28.35%, 24.22%, 23.87%, 18.72%, 15.99%, and 10.54%. Specifically, for S_0-2-3_ and S_0-3-3_, FRLD exhibited a noticeable decline in the range of x = 25~35 cm, with weak areas spanning the 10~60 cm soil layer. For S_1-2-3_ and S_1-3-3_, FRLD also showed an obvious decline in the range of x = 20~30 cm, but S_1-2-3_ exhibited dense distribution in the 30~40 cm soil layer, while S_1-3-3_ exhibited dense distribution in 20~30 cm depth. For the CK treatment, the weak areas were mostly distributed above 40 cm depth in x = 0~20 cm and below 40 cm depth in x = 20~50 cm, whereas the weak areas for S_2-2-3_ and S_2-3-3_ were both below 30 cm depth.

## 3. Discussion

### 3.1. Effects of Salt Tolerance Training on Biomass Allocation and Yield Formation of Summer Maize under Brackish Water Irrigation

Numerous studies indicate that soil salt stress affects crop canopy development, biomass accumulation, and allocation processes [17,27,28]. Among these, changes in leaf area are closely associated with crop growth and yield. Typically, a reduction in leaf area index (LAI) diminished light interception by crops, leading to a decrease in biomass production [29]. In this study, regardless of the extinction from growth stages, maize experienced a reduction in LAI upon the first occurrence of salt stress. Subsequent growth was then inhibited for a certain stage, and the extent of this inhibition correlated positively with the degree of brackish water mineralization. This was attributed to salt stress inducing ionic imbalance and nutrient disruption in crops, damaging root water uptake and leaf photosynthesis, ultimately leading to retarded growth and development [30,31,32]. Furthermore, as the brackish water mineralization degree increased, the accumulation of salts in the soil also intensified [33], resulting in a more pronounced stress response. Nevertheless, treatments exposed to the S1 and S2 levels during the first salt-tolerance-training (FSTT) stage (DAS = 21~28) exhibited less LAI suppression and greater biomass increase during the second salt-tolerance-training (SSTT) stage (DAS = 35~45), compared to untreated ones. This indicated that the FSTT enhanced maize salt tolerance, with the S2 level showing more effective conditioning. After the SSTT stage, the treatments subjected to the S1 level during the FSTT stage began to exhibit stronger growth ability. Particularly, the S_1-2-3_ treatment showed higher peak value of LAI and biomass accumulation, compared to CK. In other studies, it has also been found that irrigating with slightly brackish water in a reasonable manner did not inhibit canopy growth or biomass accumulation in crops [34,35]. 

However, the adverse consequences of brackish water irrigation should not be underestimated. In this study, the grain yields of all treatments irrigated with brackish water were lower than that of the CK treatment. Furthermore, under the same initial salt level during the FSTT stage, a higher salinity during the SSTT stage resulted in even lower maize grain yield. This trend aligned with the findings of Zhu’s experiment [36]. The decrease in grain yield was primarily attributed to a reduction in grain number per ear (Table 1). This reduction could be due to the elevated Na^+^/K^+^ ratio in summer maize leaves caused by salt tolerance training, accelerating leaf senescence and death during the reproductive growth stage [37]. Thus, CO_2_ absorption by maize was reduced, leading to decreased organic compound synthesis. This also indicated that salt tolerance training only mitigated the adverse impact of salt stress on summer maize growth to a certain extent.

In addition, the S_0-2-3_ and S_0-3-3_ treatments, which were irrigated with freshwater during the early growth stage and brackish water during the middle and later growth stages, exhibited slower rates of leaf senescence. This allowed them to undergo compensatory growth after DAS = 66. A similar phenomenon was observed in Ma’s research [24]. However, the biomass accumulation and grain yield of S_0-2-3_ and S_0-3-3_ were lower than those of the other treatments at harvest. This could be attributed to the delayed salt tolerance training, leading to lower level of leaf development when entering the tasseling stage (DAS = 52), thereby reducing photosynthetic efficiency during the critical stage of yield formation and resulting in substantial yield reduction. Consequently, conducting salt tolerance training during the early growth stage of maize is highly necessary.

### 3.2. Effects of Salt Tolerance Training on the Multidimensional Root Distribution of Summer Maize under Brackish Water Irrigation

The morphological structural characteristics of the plant root system in the three-dimensional soil space play an important role in the absorption of water and nutrients by roots, as well as the growth of the above-ground parts [38]. However, research on the response patterns of the three-dimensional root distribution under salt stress are relatively limited. Relevant studies have been conducted on small scales, focusing on plants like Arabidopsis in controlled indoor environments [39]. In the real-world context of salt-affected fields, there is still a scarcity of reports regarding the multidimensional root distribution traits exhibited by vital salt-tolerant crops such as maize, cotton, and sunflower under varying saline conditions. Therefore, investigating the growth and distribution of maize root systems in the multidimensional soil zone is of great significance for conserving freshwater resources and ensuring high maize grain yields.

**Figure 7:**
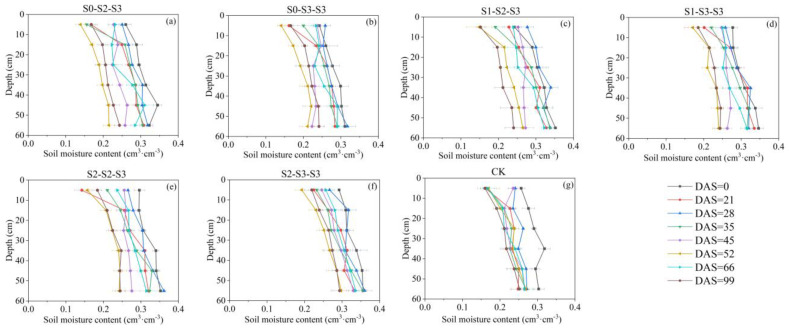
Changes in soil moisture content of each treatment during the entire growth period. The data are averaged measurements (n = 4), and horizontal bars indicate standard deviation. The S0, S1, S2, and S3 represent different salt concentration levels of irrigation water, corresponding to none (0.08 g·L^−1^), mild (2.0 g·L^−1^), moderate (4.0 g·L^−1^), and severe (6.0 g·L^−1^) stress. DAS is the abbreviation for days after sowing.

**Figure 8:**
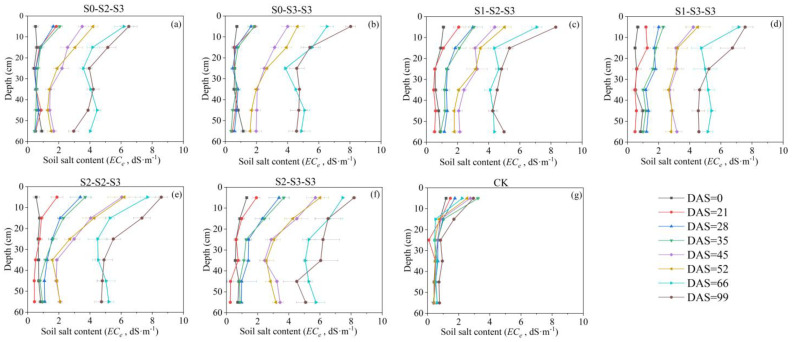
Changes in soil salt content (*EC_e_*) of each treatment during the entire growth period. The data are averaged measurements (n = 4), and horizontal bars indicate standard deviation. The S0, S1, S2, and S3 represent different salt concentration levels of irrigation water, corresponding to none (0.08 g·L^−1^), mild (2.0 g·L^−1^), moderate (4.0 g·L^−1^), and severe (6.0 g·L^−1^) stress. DAS is the abbreviation for days after sowing.

Wu et al. [40] found that under field conditions, 50~80% of maize roots were distributed in 0~20 cm depth. In this study, similar results were obtained, with the majority of FRLD for all treatments (over 65% at DAS = 66) concentrated in 0~20 cm depth throughout the entire growth period. The growth of plant roots is influenced by multiple factors, including gravitropism, hydrotropism, and oxytropism [41,42]. In this study, based on Figure 4, the CK, S_0-2-3_, and S_0-3-3_ treatments indicated that conventional freshwater irrigation and early-stage freshwater irrigation resulted in the concentration of fine root growth in the surface layer (0~10 cm depth) during the crop cycle, whereas the FSTT resulted in more fine roots growing in the deeper layer (10~20 cm depth). Considering Figure 7 and Figure 8, this phenomenon was attributed to the fact that during the FSTT stage (DAS = 21~28), the salt content in the surface layer was excessively high, while the moisture content and salt content in the 10~20 cm soil layer were within a suitable range for root growth. Nevertheless, under S_2-2-3_ and S_2-3-3_, the fine roots shifted their concentrated growth area back to the surface layer after the SSTT stage (DAS = 35~45), which might be due to the S2 level training during the FSTT stage which enhanced the adaptability of fine roots to severe salt stress and gradually encouraged their growth back into the regular area.

Relevant research has shown that fine roots have plasticity and can rapidly respond to various stressors in the soil by altering their own length, growth direction and other characteristics [43,44]. Yang et al. [45] conducted research on the two-dimensional root distribution of Jerusalem artichoke under different saline conditions. It was found that there were no significant differences in the root length density (RLD) of Jerusalem artichoke in both horizontal and vertical distributions under low saline condition. Particularly, their moderate salinity level (1.6~1.8 g salt/kg soil) even exhibited the potential to stimulate RLD growth, which was also found in our study on summer maize irrigated with brackish water. However, the two-dimensional distribution of RLD in Yang’s study was greatly changed when the salt level further increased. For example, at high salinity level (2.3~3.0 g salt/kg soil), the horizontal RLD was observed to surpass the vertical RLD. In the present study, when summer maize entered the tasseling stage (DAS = 52), the FRLD of S_2-2-3_ and S_2-3-3_ showed denser distribution both horizontally and vertically compared to the other treatments. Thereafter, at silking (DAS = 66), these two treatments still maintained dense distributions in the 0~30 cm soil layer, but more weak areas (FRLD < 0.66 cm^−3^) were found below 30 cm depth (Figure 6e,f). Particularly, the FRLD of S_2-3-3_ generally showed a decline trend compared to that at DAS = 52, indicating that the root system of summer maize had reached its maximum before the reproductive growth stage under this training mode. Referring to Figure 8, it could be observed that S_2-3-3_ reached an average *EC_e_* of 3.70 dS·m^−1^ in 0~60 cm depth at DAS = 45, which was higher than the other treatments. Thus, we speculated that the root system of S_2-3-3_, under continuous high salt stress, underwent extensive growth early to maintain water and nutrient absorption. After tasseling, its root system had already met the requirements for reproductive growth, leading to a slowdown in its development. 

On the other hand, among all treatments, only S_0-2-3_ and S_0-3-3_ generated horizontal weak areas in x = 25~35 cm which vertically spanned from 10 to 60 cm depth (Figure 6a,b), and after DAS = 66, their FRLD declining rates were lower than those of the other treatments. This indicated that experiencing salt stress for the first time at ten-leaf stage could delay fine root adaptation and reduce the limit of root extension in the horizontal direction, leading to the majority of roots accumulating in the soil close to the plants. For S_1-2-3_ and S_1-3-3_, their average FRLD of the profile (0~60 cm) did not differ greatly from CK at DAS = 52 and 66, but their weak areas of FRLD took up less space than CK in the three-dimensional soil zone, especially for S_1-2-3_, which was the least among all treatments. Considering the above-ground growth indicators, it could be inferred that these two irrigation modes, especially the S_1-2-3_ treatment, promoted a reasonable spatial distribution of FRLD under salt tolerance training. 

### 3.3. The Regulation Strategy of Root and Shoot Growth and Salt Tolerance Enhancement in Summer Maize under Brackish Water Irrigation 

The ability of plants to survive and produce harvestable yield under salt stress is known as salt tolerance [12]. The salt tolerance varies among different plant species. Generally, plant growth is inhibited under salt stress, and the stronger the salt tolerance of a plant, the less growth inhibition it experiences under salt stress [46]. Some studies have indicated that plant root growth in the early stages could be severely restricted by root zone salinity [47,48]. Our present study verifies this conclusion, demonstrating that salt tolerance training at S1 and S2 levels during the early growth stage would reduce the FRLD of summer maize, with the reduction degree positively correlated with the degree of brackish water mineralization, consistent with the growth pattern of the above-ground parts. The results of S_0-2-3_ and S_0-3-3_ treatments provided evidence that this pattern was similarly applicable to summer maize when it entered ten-leaf stage. Additionally, our study observed that after the first exposure to salt stress, the above-ground parts of all treatments could experience rapid growth during a subsequent period. This could be explained as a result of increased salt tolerance, in which the irrigation with brackish water enhanced the salt tolerance of summer maize, thus alleviating mid-to-late term salt stress and promoting leaf development and biomass accumulation. For S_2-2-3_ and S_2-3-3_, this alleviating effect became evident from the ten-leaf stage; for S_1-2-3_ and S_1-3-3_, it became apparent from the tasseling stage. However, for S_0-2-3_ and S_0-3-3_, the relief from salt stress in summer maize only became evident after the silking stage, while irreversible damages had already occurred.

The summer maize under S_0-2-3_, S_0-3-3_, S_2-2-3_, and S_2-3-3_ experienced rapid below-ground growth ahead of the above-ground parts, indicating that when crops were subjected to salt stress, new biomass would be preferentially allocated to the root system to ensure water and nutrient absorption. Similar conclusions have also been reached by Mound and Maghsoudi [22] and Meloni et al. [49]. Thus, our study suggested that increasing fine root growth could clearly enhance the salt tolerance of summer maize. However, S_1-2-3_ and S_1-3-3_ exhibited a different pattern. Their FRLD did not have a rapid growth like the above-ground parts, yet grain yield and other growth indicators performed better compared to other treatments irrigated with brackish water. As mentioned in the previous section, the spatial distributions of FRLD in S_1-2-3_ and S_1-3-3_ were more reasonable, which might be the reason behind this phenomenon. Related research has pointed out that a well-structured distribution and spatial configuration of crop’s fine roots contributed to the efficient uptake and utilization of water and nutrients by plants [50,51]. Moreover, in the presence of stress factors such as drought and salinity, plants were able to respond to such stressors by adjusting the spatial distribution of roots throughout the soil profile using their inherent root plasticity [43,44]. This enabled them to enhance their survival capability under stressful conditions. Another interpretation of this result was that appropriate salt tolerance training enhanced the root vitality of summer maize, leading to improved water uptake capacity of the root system at the same length. As a result, the plants were able to maintain water and nutrient absorption without the need for increasing soil-root contact areas by extensive fine root growth. The study by Wang [52] provided evidence for this viewpoint, showing that root vitality increased with higher salinity. Further research and investigation are necessary to explore the relationship between root vitality and brackish water irrigation under salt tolerance training.

## 4. Materials and Methods

### 4.1. Experimental Site Description

**Figure 9:**
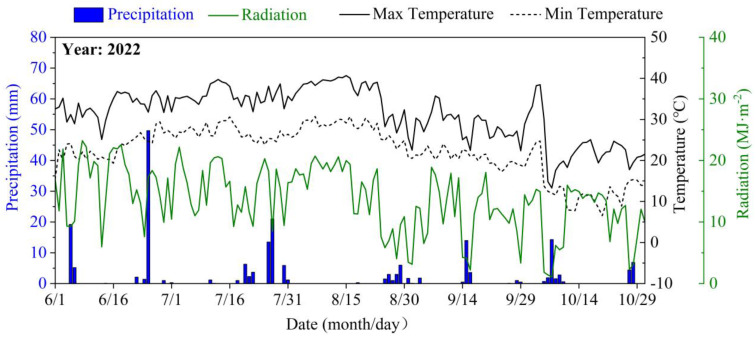
Variation in precipitation, radiation, and temperature.

The experiment was conducted from June 2022 to October 2022 in the experimental field of Jiangning Water-saving Park, Jiangsu Province, China (31°54′ N, 118°46′ E). The experimental site belongs to a subtropical monsoon climate with distinct seasons, characterized by cold winters and hot summers. The annual average temperature is 15.3 °C, with an annual sunshine duration of 2213 h and an average annual rainfall of 1051 mm. During the experiment, the average max temperature was 33.61 °C, the average min temperature was 24.96 °C, and the average radiation was 13.74 MJ·m^−2^·day^−1^ (Figure 9). Before the experiment commenced, the basic properties of the experimental soil at depths ranging from 0 to 60 cm were determined (Table 2).

### 4.2. Experimental Design

The experiment was conducted in seven micro-plots within the experimental site. Prior to the experiment, impermeable membranes were laid at the bottom and surrounding areas of each micro-plot to prevent water exchange between adjacent micro-plots and the groundwater. The dimension of each micro-plot is 2.0 m × 2.6 m × 0.7 m (length × width × depth), with partitions used in the middle to separate each micro-plot into two replicated sub-plots. The tested summer maize variety was Su Yu 29. When the moisture conditions in the plow layer were suitable, manual hill-dropping was conducted with a row spacing of 50 cm and a plant spacing of 25 cm. The maize was sown on 28 June 2022, with 3 seeds per hill and 1 seedling retained per hill after thinning, resulting in a planting density of 83,000 plants·ha^−1^. Additionally, maize harvesting took place on 5 October 2022, which was 99 days after sowing (DAS = 99).

The experiment involved the irrigation of maize at different growth stages with brackish water of varying salt levels. Four levels of salt concentration were set for irrigation water: non-stress (S0, 0.08 g·L^−1^), mild salt stress (S1, 2.0 g·L^−1^), moderate salt stress (S2, 4.0 g·L^−1^), and severe salt stress (S3, 6.0 g·L^−1^). The brackish water was prepared by mixing NaCl, Na_2_SO_4_, and NaHCO_3_ in a mass ratio of 0.61:0.31:0.08, based on the ionic composition and concentration of local brackish water. The irrigation was performed while summer maize entered the six-leaf stage, the ten-leaf stage, and the tasseling stage, forming seven different salt tolerance training modes. As shown in Table 3, the first salt-tolerance-training (FSTT) started on 21 DAS when summer maize entered the six-leaf stage and ended on 28 DAS; the second salt-tolerance-training (SSTT) started on 35 DAS when summer maize entered the ten-leaf stage and ended on 45 DAS; the severe stress test (SST) started on 52 DAS when summer maize entered the tasseling stage and ended in the silking stage (66 DAS). Overall, the experiment consisted of seven training modes, including six modes of mixed brackish and fresh irrigation water, which were S0-S2-S3, S0-S3-S3, S1-S2-S3, S1-S3-S3, S2-S2-S3, S2-S3-S3, abbreviated as S_0-2-3_, S_0-3-3_, S_1-2-3_, S_1-3-3_, S_2-2-3_, S_2-3-3_, along with the control (CK) mode of freshwater irrigation.

Prior to sowing, each micro-plot was plowed and fertilized with compound fertilizer as the base fertilizer. The effective contents of the fertilizer included 135 kg·ha^−1^ of nitrogen (as pure N), 75 kg·ha^−1^ of phosphorus (as pure P_2_O_5_), and 90 kg·ha^−1^ of potassium (as pure K_2_O), with consistent fertilization amounts across different treatment plots. After fertilization, each micro-plot was thoroughly irrigated. When summer maize reached the ten-leaf stage, an additional 135 kg·ha^−1^ of nitrogen (as pure N) was applied as topdressing, using urea as the fertilizer. In addition, various cultivation management practices such as pest control and weed control were consistent with actual production. As the experiment was conducted under rain-shelter conditions, a rain shelter was used to protect against rain on rainy days, and it was opened on non-rainy days. Therefore, the influence of rainfall was not considered.

### 4.3. Data Collection

#### 4.3.1. Soil Data

(1) Soil Moisture Content

In this experiment, soil samples were collected using a stainless steel soil auger in six layers at depths of 0~10 cm, 10~20 cm, 20~30 cm, 30~40 cm, 40~50 cm, and 50~60 cm. Each replicate plot was sampled twice, and the soil samples were placed in aluminum boxes and oven-dried at 105 °C in the laboratory to measure the soil moisture content. Sampling was conducted on 21, 28, 35, 45, 52, 66 and 99 DAS. The results are shown in Figure 7.

(2) Soil Salt Content

After the determination of soil moisture content, the soil samples were ground, and soil extracts were prepared at a soil-to-water ratio of 1:5 [53]. The soil extracts were shaken for 1 h and then allowed to settle. After the solution was clarified, the electrical conductivity (*EC_1:5_*, dS·m^−1^) was measured using a conductivity meter. The measured *EC_1:5_* value was then converted to the electrical conductivity of a saturated-paste extract (*EC_e_*, dS·m^−1^) using the empirical formula [54]. The results are shown in Figure 8. The empirical formula is given by:

(1) $$\begin{array}{c} {EC}_{e}=7.4\;\times {\;EC}_{1:5} \end{array}$$

#### 4.3.2. Crop Growth Parameters

(1) Leaf Area Index

In this experiment, the leaf area of summer maize was determined using the conventional method, which involved measuring the area of each leaf and summing up the total leaf area of the whole plant [55]. The formula for calculating the area of a single leaf is as follows:

(2) $$\begin{array}{c} Leaf\;area\;=\;Maximum\;leaf\;length\times Maximum\;leaf\;width\;\times \;0.75 \end{array}$$

Four maize plants were marked in each micro-plot for each treatment, with two plants in each replicate plot. Leaf area measurements were conducted on 11, 18, 21, 28, 35, 38, 43, 48, 53, 58, 63, 66, 76, 86, and 96 DAS, and the leaf area index (LAI) was calculated using the formula. The LAI represents the ratio of total leaf area to land area on a unit land area [56], and its calculation formula is as follows:

(3) $$LAI=\frac{leaf\;area\;per\;plant\;\times \;\;Number\;of\;plants\;per\;micro-plot}{micro-plot\;area}$$

(2) Biomass Accumulation

At the beginning and ending of each brackish water irrigation stage, namely on 21, 28, 35, 45, 52, and 66 DAS, two representative maize plants with good growth were destructively sampled from each replicate plot. The various plant organs, including leaves, stems, and ears, were oven-dried at 75 °C until they reached constant weights in order to obtain their biomasses. Among them, two plants were selected for destructive sampling on 66 DAS from the four designated maize plants. At harvest, which was 99 DAS, the remaining designated maize plants were subjected to the same treatment.

(3) Grain Yield

At harvest, grain yield measurements were conducted on all remaining maize plants within hundred-grain weight, grain number per ear, and other indicators.

#### 4.3.3. Maize Root System Parameters

In this experiment, root sampling was conducted on 21, 28, 35, 45, 52, 66, and 99 DAS. As shown in Figure 3, a root auger of 6.4 cm diameter was used to drill in layers from 0 to 60 cm depth to mainly sample the crown, primary, and seminal roots, with the maize plant as the center. For lateral root sampling, another root auger of 4.6 cm diameter was used at different distances from the maize plants. The sampling depth was consistent for all samples: 0~10 cm, 10~20 cm, 20~30 cm, 30~40 cm, 40~50 cm, 50~60 cm. Meanwhile, the maize roots below 60 cm depth had been proven to be scarce [57]. The obtained root and soil samples were separated and washed. The roots were scanned using the Epson Perfection 4990 Photo scanner, and the WinRHIZO software was used to analyze the scanned images to obtain root length and other data. Only the length of fine roots (diameter ≤ 2 mm) was taken into account in this study. After scanning, the maize roots were oven-dried at 75 °C until a constant weight was reached. The dried roots were weighed using an analytical balance with a resolution of 0.0001 g.

### 4.4. Data Analysis

The data obtained in this experiment were processed using Excel 2016 (Microsoft Corp., Redmond, WA, USA). The results of each treatment were analyzed using SPSS statistics 25 (SPSS Inc. IBM Corp., Armonk, NY, USA). Graphs were generated using Origin 2021 (OriginLab Corp., Northampton, MA, USA). The comparison of means for each treatment was conducted using the Duncan test at a significance level of *p* < 0.05.

## 5. Conclusions

Our study indicated that different salt tolerance training modes significantly affected summer maize growth and altered the temporal and spatial distribution of fine roots under brackish water irrigation. Irrigated with brackish water in the first or second salt-tolerance-training (FSTT or SSTT) stage, it could stimulate the adaptive mechanism of maize roots and promote rapid growth of above-ground parts during a subsequent period. The distinction was that appropriate salt tolerance training (S_1-2-3_ and S_1-3-3_) could promote a reasonable root spatial distribution while maintaining a relatively stable FRLD, improving leaf development and biomass accumulation, and minimizing the adverse effects of salt stress on grain yield. On the other hand, improper salt tolerance training could lead to accelerated root senescence (S_2-2-3_ and S_2-3-3_) or delayed fine root adaptation (S_0-2-3_ and S_0-3-3_), inhibit the development of above-ground parts, and reduce yield formation during the reproductive growth stage. Among all the training modes, the S_1-2-3_ treatment showed a 5.02% increase in leaf area, a 2.88% increase in above-ground biomass accumulation, and only a 5.98% decrease in grain yield, compared to conventional freshwater irrigation (CK). This suggests that irrigating the summer maize according to S_1-2-3_ training mode can utilize brackish water resources rationally with minimal yield reduction. Overall, our study provides a valuable reference for exploring the water-saving potential by more efficient utilization methods of brackish water in salt-affected areas.

## Figures and Tables

**Table 1:** Grain yield of summer maize under brackish irrigation.

Treatments	Grain Number per Ear	Hundred-Grain Weight (g)	Single Plant Grain Yield (g·Plant^−1^)	Grain Yield (kg·ha^−1^)
S0-S2-S3 (S_0-2-3_)	258.0 ± 35.1 ^bc^	32.64 ± 0.69 ^ab^	84.23 ± 11.44 ^b^	6991.44 ± 949.52 ^b^
S0-S3-S3 (S_0-3-3_)	247.2 ± 16.3 ^c^	33.48 ± 2.27 ^a^	82.75 ± 5.46 ^b^	6867.99 ± 453.18 ^b^
S1-S2-S3 (S_1-2-3_)	296.7 ± 30.0 ^abc^	32.85 ± 1.55 ^ab^	97.45 ± 9.86 ^ab^	8088.59 ± 818.38 ^ab^
S1-S3-S3 (S_1-3-3_)	295.0 ± 56.0 ^abc^	32.11 ± 1.03 ^ab^	94.74 ± 17.97 ^ab^	7863.14 ± 1491.51 ^ab^
S2-S2-S3 (S_2-2-3_)	301.8 ± 33.6 ^abc^	30.57 ± 0.36 ^bc^	92.27 ± 10.26 ^ab^	7658.35 ± 851.58 ^ab^
S2-S3-S3 (S_2-3-3_)	315.1 ± 41.9 ^ab^	29.03 ± 0.53 ^c^	91.48 ± 12.16 ^ab^	7592.82 ± 1009.28 ^ab^
CK (S_0-0-0_)	325.1 ± 51.5 ^a^	31.89 ± 1.73 ^ab^	103.66 ± 16.43 ^a^	8603.51 ± 1363.69 ^a^

The data are the means of the ears produced by the remaining 24 maize plants in each treatment at harvest. The number after the plus or minus sign represents the standard deviation. Different lowercase letters represent significant differences in grain yield. The S0, S1, S2, and S3 represent different salt concentration levels of irrigation water, corresponding to none (0.08 g·L^−1^), mild (2.0 g·L^−1^), moderate (4.0 g·L^−1^), and severe (6.0 g·L^−1^) stress.

**Table 2:** Basic properties of the experimental soil at 0~60 cm depth.

Depth	Bulk Density	Total Nitrogen	Organic Carbon	Alkali- Hydro Nitrogen	Available Phosphorus	Available Potassium	pH
cm	g·cm^−3^	g·kg^−1^	g·kg^−1^	mg·kg^−1^	mg·kg^−1^	mg·kg^−1^	
0~10	1.34	0.69	4.1	79.7	14.6	156	7.14
10~20	1.37	0.68	4.2	68.0	11.5	125	7.36
20~30	1.42	0.64	3.6	57.5	10.8	147	7.51
30~40	1.48	0.67	4.1	49.8	14.9	160	7.31
40~50	1.51	0.65	2.2	46.4	12.7	163	7.40
50~60	1.55	0.66	3.3	45.1	11.4	173	7.53

**Table 3:** Experimental design of salt concentration levels, stages of action, and duration in each micro-plot.

The Salt-Tolerance-Training Modes	Salt Concentration (g·L^−1^)							
	First Salt-Tolerance-Training (FSTT)		Recovery Stage	Second Salt-Tolerance-Training (SSTT)		Recovery Stage	Severe Stress Test (SST)	
	Initial Stage	Duration		Initial Stage	Duration		Initial Stage	Duration
	Six-Leaf Stage	DAS ^1^ = 21–28		Ten-Leaf Stage	DAS = 35–45		Tasseling Stage	DAS = 52–66
S0-S2-S3 (S_0-2-3_)	0		0	4.0		0	6.0	
S0-S3-S3 (S_0-3-3_)	0		0	6.0		0	6.0	
S1-S2-S3 (S_1-2-3_)	2.0		0	4.0		0	6.0	
S1-S3-S3 (S_1-3-3_)	2.0		0	6.0		0	6.0	
S2-S2-S3 (S_2-2-3_)	4.0		0	4.0		0	6.0	
S2-S3-S3 (S_2-3-3_)	4.0		0	6.0		0	6.0	
CK (S_0-0-0_)	0		0	0		0	0	

^1^ DAS means days after sowing.

## Data Availability

Data is contained within the article.
